# The Effects of Subalpine Forest Succession on Soil Fungal Community Composition and Diversity Vary with Soil Depth and Trophic Mode on the Eastern Qinghai–Tibetan Plateau

**DOI:** 10.3390/jof11120881

**Published:** 2025-12-12

**Authors:** Miao Chen, Jian Chen, Xiaoqiang Tang, Shun Liu, Hongshuang Xing, Xuhua Li, Lei Cai, Zhengjingru Xu, Wenhao Miao, Xia Hu, Qiuhong Feng

**Affiliations:** 1 Ecological Conservation, Restoration and Resource Utilization on Forest and Wetland Key Laboratory of Sichuan Province, Sichuan Academy of Forestry, Chengdu 610081, China; chenmiaocc@163.com (M.C.);; 2 Sichuan Giant Panda National Park Observation and Research Station, Wolong Forest Ecology Observation and Research Station of Sichuan Province, Aba 623006, China; 3 Key Laboratory of Forest Ecology and Environment of National Forestry and Grassland Administration, Ecology and Nature Conservation Institute, Chinese Academy of Forestry, Beijing 100091, China; cafchenjian@163.com (J.C.); liushun89@163.com (S.L.);; 4 Sichuan Miyaluo Forest Ecosystem Observation and Research Station, Aba 623100, China; 5 Aba Tibetan and Qiang Autonomous Prefecture Institute of Ecological Protection and Development, Aba 623006, China; 6 Department of Mathematics and Physics, Zibo Normal College, Zibo 255130, China

**Keywords:** soil fungi, community composition, diversity pattern, trophic mode, soil pH, soil moisture, soil nutrients, forest succession

## Abstract

Soil fungi play an indispensable role in maintaining soil ecosystem functions. However, how forest succession and soil depth interactively shape fungal community composition and diversity remains poorly understood. To address this, we investigated fungal communities across four successional stages and two soil depths (0–10 cm and 40–60 cm) in a subalpine forest on the eastern Qinghai–Tibetan Plateau using Illumina high-throughput sequencing. Results showed that the soil fungal community composition of different trophic modes varied significantly with both succession and soil depth. The α-diversity of symbiotic and saprotrophic fungi responded to succession in a depth-dependent manner, while β-diversity across all trophic modes was primarily driven by species turnover. Soil properties and vegetation factors collectively explained 69.85–82.91% of the variation in soil fungal community composition, with their effects being dependent on both soil depth and trophic mode. Specifically, in topsoil, the β-diversity of symbiotic fungi was influenced only by soil property heterogeneity, whereas that of saprotrophic and pathogenic fungi was shaped by both vegetation and soil property heterogeneity. In subsoil, symbiotic fungal β-diversity was co-regulated by vegetation and soil properties heterogeneity, while saprotrophic fungal β-diversity was driven solely by soil properties heterogeneity. This study demonstrates that soil depth modulates the successional dynamics of soil fungal communities and highlights the trophic-dependent drivers of fungal assembly in forest soils.

## 1. Introduction

Soil fungi, fundamental components of terrestrial ecosystems and key regulators of ecosystem processes, drive organic matter decomposition, nutrient mineralization, and plant-microbe interactions through diverse trophic strategies such as saprotrophic, symbiotrophic and pathotrophic modes [1,2,3]. They act as primary decomposers of recalcitrant compounds like lignin and cellulose, with trophic modes including saprotrophs, symbiotrophs (e.g., ectomycorrhizal and arbuscular mycorrhizal fungi) and pathogens, thus orchestrating carbon sequestration, nitrogen cycling and plant community dynamics [4,5,6]. Owing to this functional versatility, they serve as vital indicators of ecosystem health and resilience under environmental change [7,8].

Forest succession reshapes the soil environment through shifts in plant community composition, litter quality, and soil properties (e.g., pH, organic carbon, and nitrogen content), thereby imposing strong selective filters on soil fungal communities [9,10,11]. A central paradigm in successional ecology is the shift in fungal life-history strategies and trophic modes. Early successional stages, rich in labile carbon, are often dominated by ruderal Ascomycota (r-strategists), while late stages, with complex organic matter, favor competitive Basidiomycota (K-strategists) and an increased abundance of ectomycorrhizal fungi supporting climax tree species [11,12,13]. However, the trajectories of specific fungal guilds are biome-dependent and not universally predictable. For example, while Basidiomycota may increase over successional decades in tropical forests, α-diversity can peak at intermediate successional stages in subtropical systems [12,14]. These divergent patterns underscore that the assembly of fungal communities is driven by the interplay of abiotic filters (e.g., soil pH and nitrogen availability) and biotic drivers (e.g., plant host identity) [15,16,17]. Yet, a critical and less studied aspect is whether these drivers act consistently across different fungal trophic modes [18,19,20].

The influence of succession is further modulated by soil depth, which creates a strong vertical gradient in resources and environmental conditions [21,22]. The topsoil, characterized by high root density and fresh organic matter inputs, typically sustains higher fungal diversity and is dominated by saprotrophs and arbuscular mycorrhizal fungi. In contrast, subsoil is resource-limited and often harbors stress-tolerant taxa like ectomycorrhizal fungi capable of exploiting recalcitrant organic matter [21,23]. Consequently, successional dynamics are often more pronounced in the topsoil, while subsoil communities may exhibit lag effects, thereby retaining legacy taxa from earlier successional stages [24,25]. Despite these advances, critical knowledge gaps remain. Few studies have concurrently examined how forest succession differentially structures fungal trophic modes (e.g., symbiotrophs vs. saprotrophs) across soil horizons. The distinct drivers governing guild-specific responses—such as carbon allocation dynamics in topsoil versus nutrient scavenging mechanisms in subsoil—are poorly quantified, which limits our ability to predict ecosystem functioning under global change.

The subalpine forest region in western Sichuan, located on the eastern Qinghai–Tibetan Plateau [26,27], constitutes a major part of China’s second-largest forest—the Southwest Forest Area—and serves as a key ecological barrier and a critical implementation zone for the “Natural Forest Protection Project”. It plays a vital role in maintaining regional ecological balance. The original forest type in this area is mainly the subalpine dark coniferous forest dominated by *Abies faxoniana*. However, from the 1950s to the 1980s, it experienced large-scale logging. The cut-over areas have undergone natural recovery succession in the sequence of “shrubland—birch broadleaf forest—birch fir and *Abies faxoniana* mixed conifer and broadleaf forest—original *Abies faxoniana* forest”, making it an ideal place for studying natural succession [27]. Although studies have reported the impact of forest succession on soil bacterial communities in this region, the understanding of soil fungal community dynamics is still lacking. More importantly, while the interactive effects of succession and soil depth have been explored elsewhere, it remains untested whether existing models of fungal community assembly hold true under the unique environmental stressors and biogeochemical constraints of the Qinghai–Tibetan Plateau. This gap is critical, especially regarding the effects of forest succession on different trophic types of soil fungi. Given the pivotal roles that saprophytic fungi play in litter decomposition and nutrient cycling, and the threat that pathogenic fungi pose to plant health, a trophic-mode-specific understanding of fungal communities is not only scientifically valuable but also essential for effective ecosystem management.

Therefore, this study selected forests at different successional stages on the eastern Qinghai–Tibetan Plateau as the research object, aiming to analyze the changes in the composition and diversity of soil fungal communities of different trophic types during the forest recovery process and to identify the key factors influencing the composition and diversity of soil fungal communities of different trophic types. We hypothesized that: (1) fungal community composition across all trophic modes would shift significantly with forest succession, but the magnitude of these shifts would be greater in topsoil than in subsoil; (2) the α-diversity of symbiotic fungi would increase with successional maturity, particularly in the topsoil, while the response of saprotrophic and pathogenic fungi would be more depth-dependent; and (3) the β-diversity of all fungal trophic groups would be primarily driven by species turnover rather than nestedness, and the relative importance of soil vs. vegetation drivers in explaining turnover would vary with soil depth. This study integrates high-throughput sequencing with soil–vegetation analysis to incorporate fungal functional ecology into successional theory, clarifying the mechanistic links between forest development and fungal functional ecology. It is of great significance for guiding the functional, and not just structural, recovery of degraded forest ecosystems on the eastern Qinghai–Tibetan Plateau [28].

## 2. Materials and Methods

### 2.1. Study Area

The study area is situated in the Miyaluo Forest Region of Li County, Aba Tibetan and Qiang Autonomous Prefecture, Sichuan Province (31°24′–31°55′ N, 102°35′–103°04′ E), within the transitional zone between the eastern Qinghai–Tibetan Plateau fold belt and the Sichuan Basin, characterized by typical alpine valley topography [29]. This region experiences a plateau mountain climate influenced by the combined effects of air masses from the Pacific Ocean, Indian Ocean, and the Qinghai–Tibetan Plateau, exhibiting cold winters and cool summers. The mean annual temperature ranges from 6 to 12 °C, with annual precipitation of 700–1400 mm concentrated predominantly between May and September (https://data.cma.cn/, accessed on 25 April 2025). Distinct vertical vegetation zonation is observed: the subalpine zone supports primary dark coniferous forests dominated by *Abies fargesii* var. *faxoniana*, accompanied by secondary broadleaf species such as *Betula albosinensis*. Above the timberline, shrub–meadow communities prevail, dominated by Ericaceae, Rosaceae, and Salicaceae shrubs, with herbaceous layers primarily comprising Polygonaceae and Poaceae species. The predominant soil types are mountain cinnamon soil and dark brown soil. Since the logging activities in the 1950s, secondary forests comprising broadleaf and coniferous–broadleaf mixed forests have developed in this area, establishing it as a representative research site for alpine ecosystem restoration studies [30].

### 2.2. Plot Setup and Soil Sampling

This study employed the spatial-over-temporal approach commonly used in previous research to infer the long-term succession dynamics of soil fungal communities. All plots were established within a defined geographical area (the Miyaluo Forest Region) to minimize macro-environmental variation. During the 2024 growing season (July to September), four vegetation types representing distinct successional stages were selected in the Miyaluo Forest Region: early-successional broadleaf forests (stand age 20–30 years), mid-successional coniferous–broadleaf mixed forests (stand age 40–50 years and 60–70 years), and late-successional dark coniferous primary forests (stand age > 120 years). For each forest type, four 20 m × 20 m plots were established. Within each plot, all trees were measured for diameter at breast height, height, and crown width. Five nested 4 m × 4 m subplots were positioned at the four corners and center of each main plot to survey shrub community characteristics (species composition, height, and coverage). Concurrently, five 2 m × 2 m subplots were established at identical positions to assess herbaceous community attributes (species composition, height, and coverage). Based on community investigation data and allometric equations, we calculated plant community diversity (Richness) and biomass (TGB). Geographical coordinates, elevation, and topographic factors were recorded for all plots. Key characteristics of forest plots across different successional stages were summarized in Table 1.

Three random sampling points were selected per plot to excavate soil profiles (60 cm depth). Stratified soil samples were collected from four layers: 0–10 cm, 10–20 cm, 20–40 cm, and 40–60 cm. The 0–10 cm and 40–60 cm layers were designated as topsoil and subsoil, respectively. Soil samples from identical layers within the same plot were homogenized and transported to the laboratory for physicochemical analyses and microbial sequencing. Undisturbed soil cores were extracted using a 5 cm diameter soil corer from each layer to determine soil bulk density (BD) and soil water content (SWC).

### 2.3. Determination of Soil Physicochemical Properties

The SWC was determined as the amount of soil mass lost before and after drying (dried to constant weight at 105 °C) as a percentage of soil dry weight. The BD was determined as the ratio of the mass of oven-dried soil to the volume of the ring knife. Soil pH was determined using a glass electrode meter (Mettler Toledo, Zurich, Switzerland) in a water–soil solution (*v*/*w*, 2.5:1). Soil organic carbon (SOC) was measured by K_2_Cr_2_O_7_–external heating method. Soil total nitrogen (TN) was measured using a Vario EL cube CHNOS Elemental Analyzer (Elementar Analysensysteme GmbH, Langenselbold, Germany). Soil total phosphorus (TP) and total potassium (TK) were determined by plasma emission spectrometer (Thermo IRIS Intrepid II XSP, Thermo Fisher Scientific, Franklin, TN, USA) using the acid dissolution method (HNO_3_-HClO_4_-HF). Soil available phosphorus (AP) was determined by continuous flow autoanalyzer (SEAL AutoAnalyzer 3, Bran and Luebbe GmbH, Norderstedt, Germany) using molybdenum blue colorimetric method after extraction with a mixed solution (0.05 M HCl and 0.025 M H_2_SO_4_). Soil available potassium (AK) was extracted with a neutral 1 mol/L ammonium acetate solution and determined by flame photometry. Soil ammonium-N (NH_4_^+^-N) and nitrate-N (NO_3_^−^-N) were measured by continuous flow autoanalyzer (SEAL Auto Analyzer 3, Bran and Luebbe GmbH, Germany) after extraction with 2 M KCl solution. The soil available nitrogen (AN) was calculated as the sum of NH_4_^+^-N and NO_3_^−^-N [26].

### 2.4. DNA Extraction, MiSeq Sequencing, and Bioinformatics

A total of 32 individual soil samples of microbial DNA were extracted using the DNeasy PowerSoil Kit (100) (QIAGEN, Hilden, Germany) following the manufacturer’s instructions. After the concentration of the extracted DNA was quantified, the fungal ITS2 genes were amplified using fITS7 (5′-GTGARTCATCGAATCTTTG-3′)/ITS4 (5′-AGCCTCCGCTTATTGATATGCTTAART-3′). The PCR 25 μL reaction system includes 8.5 μL of deionized sterile water, 0.75 μL of each primer, 12.5 μL of KAPA enzyme, and 2.5 μL of DNA template. The PCR reactions were conducted using the following program: 3 min of denaturation at 95 °C, 35 cycles of 30 s at 98 °C, 30 s for annealing at 56 °C, and 30 s for elongation at 72 °C, and a final extension at 72 °C for 10 min. Deionized sterile distilled water was also used as a negative control in all PCR amplification steps to detect the presence of contamination during the experiment. PCR products were extracted from a 2% agarose gel and further purified using the OMEGA E.Z.N.A.^®^ Cycle Pure Kit (OMEGA, Miami, FL, USA) and quantified using QuantiFluor™-ST (Promega, Madison, WI, USA). Purified PCR products were subjected to high-throughput sequencing (IlluminaMiseq PE 250, Illumina, Inc., San Diego, CA, USA) [26].

Raw sequences were demultiplexed, quality filtered by Trimmomatic (version 0.39), and merged by FLASH (version 1.2.11) [31]. The chimeric sequences were identified and removed using UCHIME (version 4.2.4) [32], the non-chimera sequences were screened for quality, and then operational taxonomic units (OTUs) were clustered with a 97% similarity cutoff using Usearch (version 11.0.667) [33]. The taxonomy of each ITS gene sequence was analyzed using the UNITE database (version 9.0) [34]; the confidence threshold was 0.65. The number of sequences per sample was normalized to the minimum sample size using the *sub.sample* command in Mothur (version 1.45.2) to remove the effect of the number of sequences across samples on the fungal community [35]. Soil fungi were divided into trophic modes by the FungalTraits (version 1.3) [36].

### 2.5. Statistical Analysis

The soil fungal community dataset was divided into three subsets based on trophic modes: the “symbiotic fungi”, “saprophytic fungi”, and “pathogenic fungi” datasets (i.e., contained the OTUs of the trophic modes symbiotroph, saprotroph, and pathotroph, respectively). The three fungal datasets (symbiotic, saprophytic, and pathogenic fungi) were analyzed separately. A Mantel test was used to check whether soil samples were independent or spatially autocorrelated [37]. The OTUs table was used for downstream community composition and diversity analysis. The relative abundance of soil fungal phyla levels and trophic modes in each sample was calculated and ranked. Analysis of similarities (ANOSIM), multi-response permutation procedures (MRPP), and permutational multivariate analysis of variance (PERMANOVA) were employed to assess differences in soil fungal community composition during forest succession [38]. The α-diversity was estimated using the Shannon–Wiener index, and a one-way analysis of variance (ANOVA) was employed to test whether forest succession had a significant impact on the α-diversity of soil fungi [39]. The β-diversity and its components (turnover and nestedness) were computed using the function *beta.pair* and *beta.pair.abund* in R package ‘betapart’ (version 1.5.4) [40].

To disentangle the relationship between soil fungal community composition and environmental variables, the distance-based redundancy analysis (db-RDA) and Monte Carlo permutation test (999 permutations) were performed. For the db-RDA analysis, the significance of a full model including all the explanatory variables was first tested, and then the model was simplified by forward-model selection using the function *ordiR2step* in R package ‘vegan’ (version 2.6-2). Hierarchical partitioning was used to decompose the generalized mixed-effect model to determine the relative importance of each variable for the variation in soil fungal α-diversity under forest succession using R package ‘glmm.hp’ (version 0.1-8), with soil properties and vegetation factors set as fixed effects and forest successional stage set as a random effect. The multiple regression on matrices (MRM) method was used to examine the relative effects of the dissimilarity in soil properties and vegetation factors (based on Euclidean distances) on β-diversity and its two components (based on Bray–Curtis index) using R package ‘ecodist’ (version 2.0.9) [41]. To identify the most parsimonious model and avoid overfitting, we employed a model selection approach based on the Akaike Information Criterion (AIC). We constructed a global model containing all environmental variables. The best-fit model was then selected through a stepwise algorithm from the R package ‘MASS’ (version 7.3-65). We report the results from the AIC-selected best model. All statistical analyses and plotting were conducted in R (v4.3.2; http://www.r-project.org/, accessed on 25 April 2025).

## 3. Results

### 3.1. Soil Fungal Community Composition

**Figure 1:**
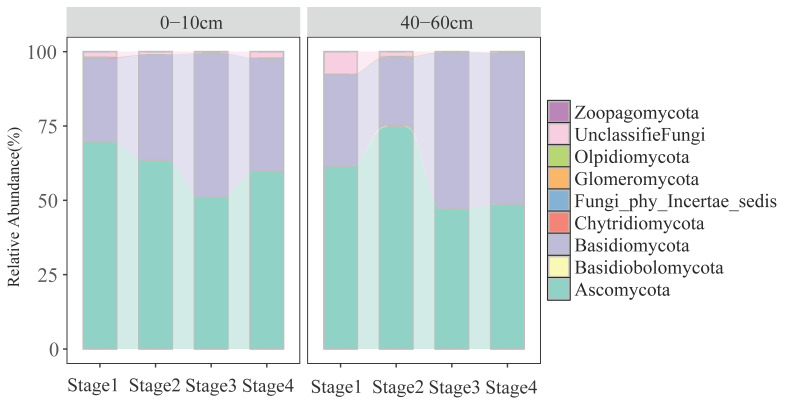
Relative abundance of the soil fungal phyla in different soil layers.

The relative abundance of dominant fungal phyla varied significantly with both soil depth and forest successional stage (Figure 1). Ascomycota and Basidiomycota were consistently the two most dominant phyla across all stages and depths. In particular, the topsoil supported a significantly higher relative abundance of Ascomycota compared to the subsoil, while Basidiomycota showed higher abundance in later successional stages. Notable shifts in phyla such as Chytridiomycota and Glomeromycota were also observed along the successional gradient, suggesting differential responses of fungal taxa to vegetation development. Subsoil communities exhibited lower overall abundance but higher compositional variability across stages, particularly in early successional phases (Figure 1).

**Figure 2:**
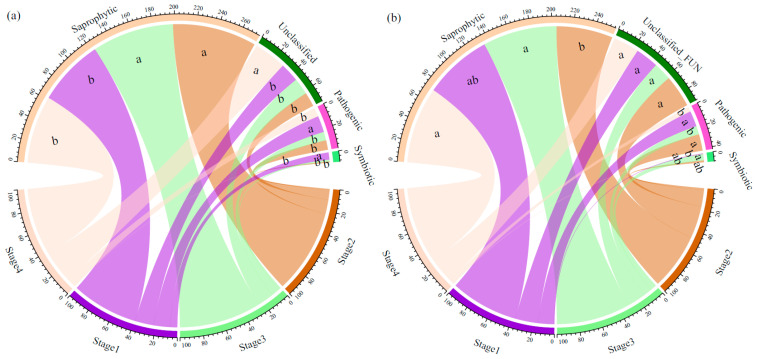
Relative abundance of soil fungi with different trophic modes in different soil layers ((**a**): 0–10 cm, (**b**): 40–60 cm). Different lowercase letters indicate significant differences in the relative abundance of soil fungi with the same trophic mode across different succession stages.

In total, 1083 OTUs (63.97% of the total OTUs) were assigned to three trophic modes. In the topsoil, the relative abundance of saprotrophic fungi at Stages 2 and 3 was significantly higher than that at Stages 1 and 4, while the relative abundance of pathogenic fungi at Stages 2, 3, and 4 was significantly lower than that at Stage 1. Additionally, the relative abundance of symbiotic fungi at Stage 1 was significantly higher than that at Stages 2, 3, and 4 (Figure 2a). In the subsoil, the relative abundance of saprotrophic fungi at Stages 3 and 4 was significantly higher than that at Stage 2. The relative abundance of pathogenic fungi at Stages 1 and 2 was significantly higher than that at Stages 3 and 4, and the relative abundance of symbiotic fungi at Stage 3 was significantly higher than that at Stage 4 (Figure 2b).

The composition of soil fungal communities significantly differed across trophic modes and soil depths during forest succession, as revealed by multiple statistical analyses (Table 2). In the topsoil, all three trophic modes exhibited significant differences in community composition: symbiotic fungi (ANOSIM: R = 0.241, *p* < 0.01), saprophytic fungi (ANOSIM: R = 0.283, *p* < 0.01), and pathogenic fungi (ANOSIM: R = 0.237, *p* < 0.05). Consistent results were obtained using MRPP and PERMANOVA (all *p* < 0.05). In the subsoil, significant differences were also detected in symbiotic fungi (ANOSIM: R = 0.286, *p* < 0.001) and saprophytic fungi (ANOSIM: R = 0.158, *p* < 0.05), supported by MRPP and PERMANOVA (all *p* < 0.05). In contrast, pathogenic fungal communities in the subsoil did not show significant differences across groups (ANOSIM: R = 0.007, *p* > 0.05; MRPP: *p* > 0.05; PERMANOVA: *p* > 0.05).

### 3.2. Soil Fungal Diversity

**Figure 3:**
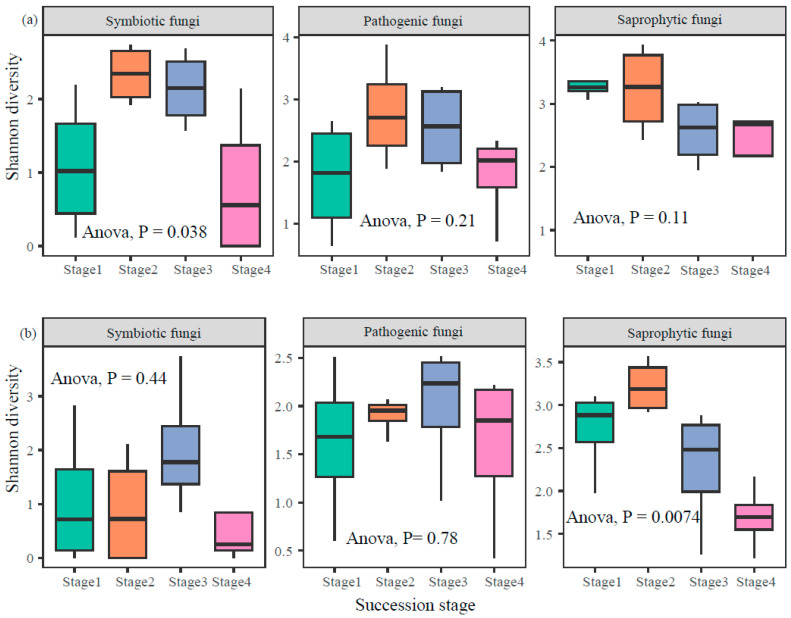
Patterns of fungal α-diversity in topsoil (**a**) and subsoil (**b**) along forest successional stages.

Soil fungal Shannon diversity exhibited distinct patterns across successional stages and soil depths, with varying responses among trophic modes (Figure 3). In the topsoil, symbiotic fungi showed a significant shift in diversity across succession (ANOVA, *p* = 0.038), while saprophytic and pathogenic fungi did not exhibit significant differences (*p* > 0.05). In contrast, within the subsoil, saprophytic fungi diversity changed significantly over successional stages (ANOVA, *p* = 0.0074), whereas symbiotic and pathogenic fungi diversity remained relatively stable (*p* > 0.05). These results suggested that soil depth modulates the successional trajectory of fungal diversity in a trophic-specific manner (Figure 3).

**Figure 4:**
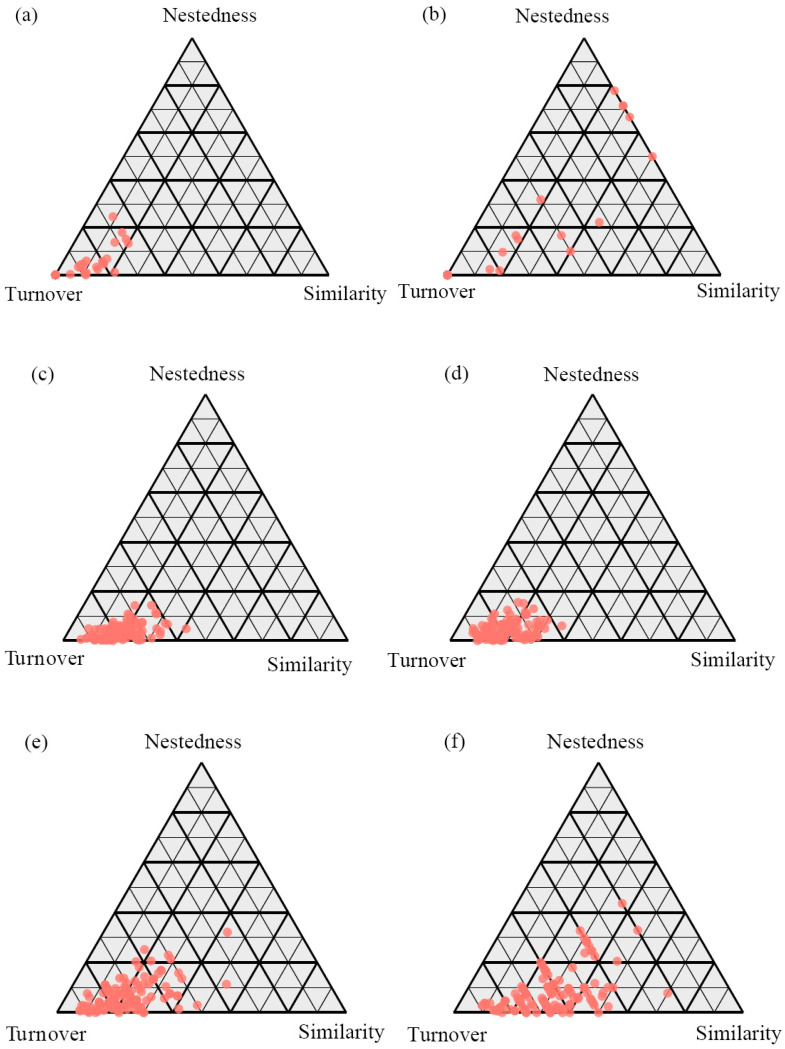
The β-diversity patterns of symbiotic fungi (**a**,**b**), saprophytic fungi (**c**,**d**), and pathogenic fungi (**e**,**f**) in topsoil (**a**,**c**,**e**) and subsoil (**b**,**d**,**f**) along forest successional stages.

The decomposition of β-diversity revealed that the turnover component was the dominant driver across all fungal trophic modes and in both soil layers (Figure 4). Notably, the relative contribution of turnover was consistently higher in topsoil than in subsoil for each fungal functional group. Specifically, symbiotic fungi in topsoil exhibited the highest proportion of turnover (98.44%), followed by saprophytic (95.56%) and pathogenic fungi (90.95%). In the subsoil, the turnover component remained predominant, with contributions of 94.37% for symbiotic, 94.82% for saprophytic, and 87.52% for pathogenic fungi. Consequently, the nestedness component showed an inverse pattern, with the highest contributions observed for pathogenic fungi (9.05% in topsoil and 12.48% in subsoil). This consistent pattern suggests that species replacement (turnover), rather than the loss or gain of species (nestedness), primarily structures the fungal communities along the forest successional gradient, with the strength of this process being most pronounced in the topsoil and for symbiotic fungi (Figure 4).

### 3.3. The Drivers of Soil Fungal Community

**Figure 5:**
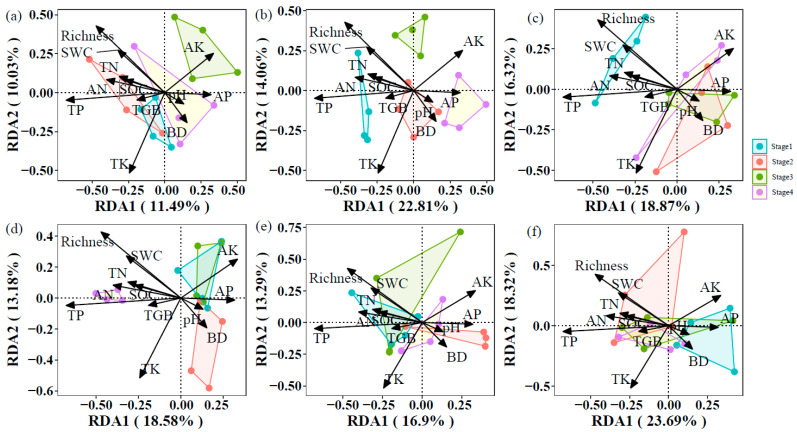
Distance-based redundancy analysis (db-RDA) demonstrated the effect of soil properties and vegetation factors on community composition of symbiotic fungi ((**a**): 0–10 cm, (**d**): 40–60 cm), saprophytic fungi ((**b**): 0–10 cm, (**e**): 40–60 cm), and pathogenic fungi ((**c**): 0–10 cm, (**f**): 40–60 cm).

In the 0–10 cm layer, soil properties and vegetation factors explained 81.53% variation in community composition of soil symbiotic fungi, in which the RDA1 and RDA2 axes explained 11.49% and 10.03%, respectively (Figure 5a). Soil properties and vegetation factors explained 78.93% and 82.91% of the variation in community composition of soil saprophytic fungi and pathogenic fungi, respectively (Figure 5b,c). In the 40–60 cm layer, soil properties and vegetation factors explained 80.67, 79.72, and 69.85% variations in community composition of soil symbiotic fungi, saprophytic fungi, and pathogenic fungi, respectively (Figure 5d–f).

The community composition of soil fungi with different trophic modes was primarily driven by distinct soil properties at different depths, not by a consistent set of factors across the profile. In the 0–10 cm layer, TN, TK, and BD significantly influenced symbiotic and pathogenic fungi, while TK significantly affected saprophytic fungi. In contrast, within the 40–60 cm layer, TK, pH, and TGB were key drivers for symbiotic fungi, whereas AK and TGB significantly affected saprophytic fungi. Pathogenic fungi composition was significantly influenced by SOC, TN, and TP in the topsoil but only by TP in the subsoil. Notably, TN exerted a stronger effect on symbiotic and pathogenic fungi than on saprophytic fungi in the 0–10 cm layer. TP significantly impacted pathogenic fungi across both depths but showed no significant effect on symbiotic or saprophytic fungi in the topsoil. TGB had a particularly strong effect on symbiotic fungi in the subsoil, while pH significantly influenced symbiotic fungi only in the deeper layer. Factors such as SWC, AN, and AP showed no consistent significant effects (Table 3).

**Figure 6:**
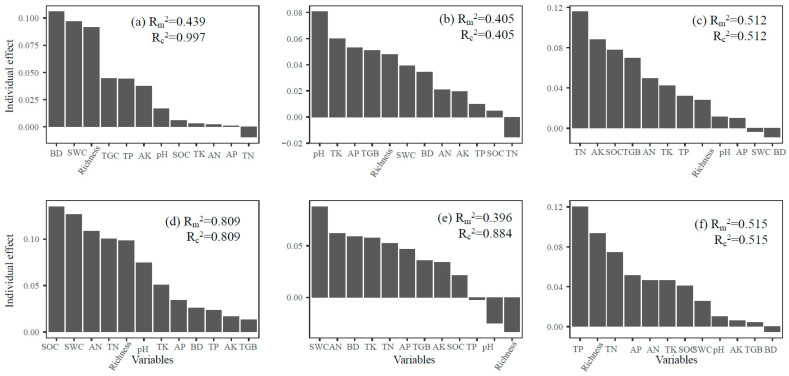
Relative importance of explanatory variables for the variation in α-diversity of symbiotic fungi ((**a**): 0–10 cm, (**d**): 40–60 cm), saprophytic fungi ((**b**): 0–10 cm, (**e**): 40–60 cm), and pathogenic fungi ((**c**): 0–10 cm, (**f**): 40–60 cm).

The variation in α-diversity of soil fungal communities across different trophic modes was explained distinctly by soil properties, with both depth and successional stage playing important roles (Figure 6). In the topsoil, environmental variables explained a substantial proportion of the diversity in symbiotic fungi (marginal R^2^ = 0.439; conditional R^2^ = 0.997). The SOC, SWC, AN, and TN were identified as key factors influencing diversity across trophic groups. For saprophytic fungi, the model accounted for 40.5% of the diversity variation, while for pathogenic fungi, explanatory power reached 51.2%. In the subsoil, the explanatory strength for symbiotic fungi was notably high (R^2^ = 0.809), with SOC, SWC, AN, and TN remaining influential. Saprophytic fungal diversity was also strongly associated with soil properties (R^2^ = 0.884), particularly TK, AK, and SOC. For pathogenic fungi, 51.5% of diversity variation was explained, with TP and TN among the most important predictors. These results highlight the depth-dependent and trophic-specific responses of soil fungal diversity to edaphic factors during forest succession.

The MRM analysis revealed that soil properties and vegetation factors significantly influenced the β-diversity of soil fungal communities, with varying effects across soil depths and fungal trophic modes (Table 4). In the topsoil, environmental variables showed the strongest explanatory power for saprotrophic fungi (R^2^ = 48.2%, *p* < 0.001), followed by pathotrophic (R^2^ = 19.3%, *p* < 0.001) and symbiotrophic fungi (R^2^ = 12.9%, *p* < 0.01). Key drivers included pH, SOC, TN, and SWC. Both turnover and nestedness components were significantly affected across all fungal groups. In the subsoil, explanatory power was generally lower. Saprotrophic fungi remained most responsive to soil factors such as AN, SWC, and TP (R^2^ = 23.2%, *p* < 0.001), while weaker but significant effects were observed for pathotrophic and symbiotrophic fungi (R^2^ = 6.0–16.7%, *p* < 0.05). Overall, fungal β-diversity and its components were more strongly influenced by soil and vegetation variables in the topsoil than subsoil, with saprotrophic fungi showing the greatest sensitivity to environmental changes (Table 4).

## 4. Discussion

### 4.1. Soil Fungal Community Composition

Our findings reveal that forest succession and soil depth function as synergistic filters, driving fungal community assembly through distinct niche-based processes (Figure 1). A key manifestation is the successional shift in topsoil from Ascomycota to Basidiomycota dominance, reflecting a fundamental transition in ecosystem function: from rapid nutrient cycling supported by r-strategist saprotrophs in early stages to the slow decomposition of recalcitrant litter by K-strategists in mature forests [42,43,44,45]. This overarching framework contextualizes guild-specific dynamics, such as the mid-succession saprotroph peak (Stages 2–3, Figure 2a), which likely represents a transient resource pulse during canopy closure before litter quality becomes increasingly recalcitrant [45]. Critically, our results establish a depth-dependent assembly model. Topsoil communities are strongly coupled to vegetation dynamics, as evidenced by significant successional shifts in both symbiotic and pathogenic fungi, highlighting the primacy of biotic interactions [46,47]. In stark contrast, the stability of subsoil pathogenic communities across succession (Table 2) demonstrates that abiotic filtering overrides vegetation influences at depth, selecting for a core community of generalist taxa [46,48]. This decoupling suggests the subsoil acts as a buffered microbial reservoir, challenging the direct application of above-ground successional theory to the entire soil profile. This peak in functional diversity likely represents a critical transitional state for the ecosystem, where concurrent high resource availability and habitat heterogeneity support a broader spectrum of ecological niches [46,47,49]. Our results demonstrated a consistent decline in the relative abundance of pathogenic fungi in both topsoil and subsoil with forest succession (Figure 2). This finding suggests that promoting natural forest succession to later stages could be an effective strategy for mitigating pathogen-related risks and enhancing overall forest health [49]. This pattern can be attributed to interconnected mechanisms associated with forest maturation: (1) the increase in tree diversity and complexity in late-successional forests may dilute host density and reduce the transmission efficiency of specialized pathogens [50] and (2) the accumulation of antifungal compounds in the soil from the recalcitrant litter of late-successional tree species (e.g., *Abies faxoniana*) creates an unfavorable chemical environment for many soil-borne pathogens [26].

### 4.2. Soil Fungal Diversity

The divergent responses of fungal trophic modes to forest succession across soil depths reflect fundamental ecological strategies shaped by resource availability and habitat constraints [14]. In topsoil, the significant shift in symbiotic fungal diversity (Figure 3a) aligns with host plant succession: early-stage herbaceous communities favor arbuscular mycorrhizal fungi (e.g., Glomeraceae), while late-stage woody dominance selects for ectomycorrhizal taxa (e.g., Russulaceae) [26,27,37]. This transition is driven by changing root exudate chemistry and nitrogen mobilization demands [27]. In contrast, saprophytic and pathogenic fungi exhibited minimal α-diversity changes in topsoil, suggesting stable access to organic substrates and host plants due to continuous litter input and root density [51]. Subsoil dynamics revealed a contrasting pattern: saprophytic fungi diversity responded strongly to succession (Figure 3b), likely due to slow leaching of complex carbon (e.g., lignin) into deeper layers during late succession [52,53]. This lagged response supports the “subsoil carbon bottleneck” hypothesis, where K-strategist saprotrophs (e.g., Agaricales) gradually replace r-strategists (e.g., Mucorales) as recalcitrant compounds accumulate [54]. The stability of symbiotic and pathogenic fungi in subsoil underscores limited root penetration and reduced host–pathogen interactions at depth [53,55,56].

The consistent dominance of species turnover across all fungal functional groups and soil layers (Figure 4) robustly demonstrates that the reassembly of fungal communities during forest succession is primarily driven by the replacement of taxa, rather than by nested species loss or gain. This overarching pattern strongly aligns with the concept of deterministic assembly, whereby successionally shifting environmental conditions—such as soil pH, organic carbon, and nutrient availability—act as a filter, selectively excluding ill-adapted taxa and favoring those with traits suited to the new habitat [57,58]. Notably, the relative contribution of turnover was systematically higher in the topsoil than in the subsoil for every fungal guild. This vertical disparity suggests that topsoil communities are subject to stronger deterministic filters or greater spatial heterogeneity linked to plant roots and organic matter inputs, leading to more pronounced species replacement along the successional gradient [59]. Furthermore, the magnitude of turnover varied significantly among fungal trophic modes. The exceptionally high turnover in symbiotic fungi (98.44% in topsoil), which closely depend on living host plants, underscores the critical role of plant community succession in driving fungal community composition. In contrast, the comparatively lower, though still dominant, turnover in pathogenic fungi (90.95% in topsoil), coupled with their highest nestedness component, may reflect a combination of high host specificity and stronger dispersal limitations that prevent complete community turnover, resulting in a degree of nested species loss [60,61]. Saprophytic fungi, with their intermediate turnover values, likely respond to both broader environmental conditions and the changing quality of organic matter substrates [62,63]. The attenuated turnover signals in the subsoil, particularly among saprotrophs (94.82% vs. 95.56% in topsoil), indicate that deeper soil layers preserve functional traits and taxa from earlier successional stages. This “ecological memory” effect could be crucial for ecosystem resilience, potentially buffering biogeochemical processes like carbon cycling during disturbance events [64]. This finding advocates for management practices that minimize soil disruption to protect this functional legacy.

### 4.3. The Drivers of Soil Fungal Community

Our analysis reveals a fundamental shift in the drivers of fungal community composition between soil layers, reflecting a depth-dependent divergence in energetic and nutrient constraints (Figure 5 and Table 3). In topsoil, the strong association between community composition of symbiotic fungi and TN stems from the high-energy-input environment. We hypothesize that plants engage in efficient carbon–nitrogen exchange with symbiotic fungi through photosynthate allocation, forming an effective nutrient acquisition mechanism [26,27]. In subsoil, community composition of symbiotic fungi was primarily driven by plant biomass and pH, indicating that host carbon allocation replaces nitrogen acquisition as the dominant limiting factor under carbon-limited conditions. pH further influences community structure by regulating nutrient solubility and metal toxicity in the acidic mineral layers [65], while the diminished role of TN may be related to nitrogen immobilization in recalcitrant organic matter at depth [52,53]. For community composition of pathogenic fungi, the driving factors shifted from AN and host plant diversity in topsoil to specialized phosphorus limitation in subsoil. We propose that in nutrient-poor subsoil, phosphorus becomes a critical factor for host invasion and pathogenicity. Generalist pathogens may exploit host plants under phosphorus-deficient conditions to infect stressed root systems [14,56]. The community composition of saprotrophic fungi exhibited a transition from TK dependence in topsoil to co-limitation by AK and TP in subsoil, corresponding to changes in substrate properties. Topsoil saprotrophs require potassium to activate lignin-degrading enzymes [51], whereas those in subsoil face a “carbon bottleneck” when decomposing recalcitrant organic matter, with their activity co-limited by phosphorus (for energy metabolism) and potassium (for osmotic balance) [66,67].

In topsoil, the soil fungal α-diversity of all trophic modes was closely associated with SOC and TN (Figure 6a–c). The strong response of symbiotic fungi to SOC aligns with the pattern of ectomycorrhizal fungi relying on particulate organic carbon for nutrient exchange [13]. The dual importance of TN and AN underscores the central regulatory role of nitrogen, constraining both enzyme production for litter decomposition (saprotrophs) and virulence factor synthesis (pathogens) [56,57]. The SWC influenced all groups by mediating oxygen diffusion and hyphal mobility [52,53]. In subsoil (Figure 6d–f), fungal α-diversity shifted toward potassium- and phosphorus-driven patterns: saprotrophs depended on TK to activate ligninolytic enzymes [67] and on AK to maintain osmotic balance [14]; pathogens required TP for ATP-dependent host invasion and TN for toxin synthesis [57,66]; and symbiotic fungi retained their carbon-to-nitrogen ratio dependence—potentially linked to deep-root carbon allocation [53,55]—but the increased influence of pH suggests a mechanism for avoiding aluminum toxicity in acidic subsoil [65]. During forest succession, initial nutrient enrichment in topsoil promoted diversity across all trophic groups [19]. However, subsoil responses diverged: saprotroph diversity became increasingly potassium-limited due to leaching of base cations [66], and pathogen diversity shifted toward phosphorus limitation, consistent with the development of phosphorus-deficient subsoil in late-successional forests [67]. These findings support the hypothesis that forest succession “unlocks” depth-dependent nutrient bottlenecks [68].

The distinct drivers of β-diversity across soil depths reveal how environmental filtering and nutrient co-limitation structure fungal communities. In topsoil, the strong pH and SOC constraints on saprotrophs reflect niche differentiation tied to their core ecological role: acid-tolerant guilds dominate decomposition under low pH, while high SOC sustains ligninolytic enzymes for processing complex organic matter [13,65]. The dominance of turnover over nestedness in their β-diversity confirms that succession acts as a strong environmental filter, replacing rather than simply subsetting decomposer taxa [69]. Conversely, the subsoil environment imposes a different selective regime, shifting saprotrophs toward ammonium and phosphorus dependence—a pattern indicative of nutrient co-limitation under the “carbon bottleneck” of recalcitrant organic matter [67,68]. Here, the continued dominance of turnover suggests that deep soil habitats foster divergent evolutionary trajectories, selecting for specialists in potassium solubilization and phosphate scavenging [65,67]. For symbiotic fungi, the strong nitrogen-linked turnover in topsoil aligns with their role in host nutrient acquisition, whereas the unexpected potassium influence may reflect its role in maintaining membrane potential during sustained plant–fungal exchange [56,70]. Pathogens’ confinement to topsoil conditions and sensitivity to moisture fluctuations further underscore how abiotic filtering overrides host specificity in structuring antagonistic interactions across the soil profile [52,53]. To further refine the findings of this study, future research should consider incorporating additional factors such as soil texture and soil fungal predators (e.g., protists and nematodes). These elements exert distinct yet critical influences on the habitat structure of soil fungi across different trophic levels and on top-down regulatory pressures, which may help elucidate the underlying mechanisms governing soil fungal community composition and diversity.

## 5. Conclusions

This study demonstrates that subalpine forest succession significantly alters soil fungal community composition and diversity on the eastern Qinghai–Tibetan Plateau, with these effects being strongly modulated by soil depth and fungal trophic mode. The successional dynamics of fungal communities were primarily driven by species turnover, reflecting continuous environmental filtering and ecological niche differentiation throughout vegetation development. Our findings highlight that the relative importance of soil properties versus vegetation factors in shaping fungal communities depends critically on both soil depth and fungal trophic modes. The depth-dependent and successional-stage-specific responses of fungal communities observed in this study have important implications for predicting how these critical soil organisms may respond to ongoing environmental changes in high-elevation ecosystems.

## Figures and Tables

**Table 1:** Basic information of forest sample plots across different successional stages.

Abbreviation	Stand Age (a)	Vegetation Types	Dominant Plant Species
Stage 1	20–30	Broadleaf forests	*Betula albosinensis*, *Acer davidii*
Stage 2	40–50	Coniferous–broadleaf mixed forests	*Betula utilis*, *Abies fargesii* var. *faxoniana*, *Picea purpurea*
Stage 3	60–70	Coniferous–broadleaf mixed forests	*Betula albosinensis*, *Abies fargesii* var. *faxoniana*
Stage 4	>120	Dark coniferous primary forests	*Abies fargesii* var. *faxoniana*, *Rhododendron* spp.

(a) As the unit of stand age (in years).

**Table 2:** Analysis of similarities (ANOSIM), multi-response permutation procedures (MRPP), and permutational multivariate analysis of variance (PERMANOVA) of soil fungal community composition across different trophic modes and soil depths during forest succession. Asterisks indicate significant level: ns, *p* > 0.05; * *p* < 0.05; ** *p* < 0.01; *** *p* < 0.001.

Soil Layers	Trophic Modes	ANOSIM	MRPP	PERMANOVA
0–10 cm	Symbiotic fungi	0.241 **	0.009 **	0.214 **
0–10 cm	Saprophytic fungi	0.283 **	0.109 ***	0.349 **
0–10 cm	Pathogenic fungi	0.237 *	0.065 ***	0.290 **
40–60 cm	Symbiotic fungi	0.286 ***	0.013 ***	0.220 ***
40–60 cm	Saprophytic fungi	0.158 *	0.065 *	0.286 *
40–60 cm	Pathogenic fungi	0.007 ns	0.004 ns	0.204 ns

**Table 3:** Results of Monte Carlo permutation tests for soil properties and vegetation factors on soil fungal community composition. Asterisks indicate significant level: ns, *p* > 0.05; * *p* < 0.05; ** *p* < 0.01; *** *p* < 0.001.

Influencing Factors	0–10 cm			40–60 cm		
	Symbiotic Fungi	Saprophytic Fungi	Pathogenic Fungi	Symbiotic Fungi	Saprophytic Fungi	Pathogenic Fungi
SOC	0.344 ns	0.110 ns	0.562 **	0.292 ns	0.460 *	0.080 ns
TN	0.444 *	0.041 ns	0.449 *	0.344 ns	0.314 ns	0.107 ns
TP	0.243 ns	0.141 ns	0.490 *	0.254 ns	0.429 *	0.429 *
TK	0.370 *	0.407 *	0.068 ns	0.444 *	0.213 ns	0.252 ns
AN	0.285 ns	0.048 ns	0.603 **	0.156 ns	0.115 ns	0.163 ns
AP	0.237 ns	0.337 ns	0.044 ns	0.422 *	0.186 ns	0.097 ns
AK	0.097 ns	0.278 ns	0.190 ns	0.322 ns	0.380 *	0.143 ns
SWC	0.245 ns	0.289 ns	0.035 ns	0.128 ns	0.056 ns	0.174 ns
BD	0.370 *	0.324 ns	0.453 *	0.031 ns	0.302 ns	0.058 ns
pH	0.059 ns	0.663 **	0.292 ns	0.636 **	0.180 ns	0.022 ns
TGB	0.087 ns	0.395 *	0.019 ns	0.799 ***	0.036 ns	0.035 ns
Richness	0.346 ns	0.277 ns	0.568 **	0.229 ns	0.056 ns	0.395 *

**Table 4:** Results of the MRM test for the distance of soil properties, vegetation factors, and β-diversity. Asterisks indicate significant level: * *p* < 0.05; ** *p* < 0.01; *** *p* < 0.001.

Soil Layer	Soil Fungi	Components	Fit Equation	R^2^
0–10	Symbiotrophic	β-diversity	0.992 − 0.0003AN +0.0034pH + 0.0008TK + 0.0022TN	0.129 **
		Turnover	0.979 − 0.0001AK + 0.0203pH − 0.0009SOC + 0.0108TN + 0.0313TP	0.146 **
		Nestedness	0.0244 + 0.0001AK − 0.0211BD − 0.0090pH + 0.0007SOC − 0.0341SWC − 0.0013TK − 0.0089TN − 0.0186TP	0.187 **
0–10	Saprotrophic	β-diversity	0.811 − 0.015AP + 0.080pH + 0.0029Richness + 0.0046SOC − 0.468SWC − 0.014TK − 0.066TN	0.482 ***
		Turnover	0.779 − 0.0004AN − 0.012AP + 0.070pH + 0.003Richness + 0.0071SOC − 0.544SWC − 0.014TK − 0.078TN	0.477 ***
		Nestedness	0.035 + 0.0001AN − 0.0019SOC + 0.073SWC + 0.017TN	0.199 ***
0–10	Pathotrophic	β-diversity	0.909 + 0.041pH + 0.001Richness − 0.120SWC + 0.006TK	0.193 ***
		Turnover	0.766 + 0.0004AK + 0.268BD − 0.0039SOC − 0.0037TGB + 0.018TK + 0.066TN	0.144 **
		Nestedness	0.100 − 0.0079AP + 0.0036TGB − 0.146TP	0.128 **
40–60	Symbiotrophic	β-diversity	0.995 − 0.0001AK + 0.0002Richness + 0.0003SOC	0.091 *
		Turnover	0.912 + 0.0958pH + 0.0047Richness − 0.0135TK + 0.0894TN − 0.311TP	0.142 **
		Nestedness	0.0813 − 0.0922pH − 0.0045Richness + 0.0132TK − 0.0862TN + 0.310TP	0.139 **
40–60	Saprotrophic	β-diversity	0.749 − 0.0012AN + 0.340SWC + 0.263TP	0.232 ***
		Turnover	0.714 − 0.0016AN + 0.312TP + 0.395SWC	0.246 ***
		Nestedness	0.027 + 0.0003AN − 0.049SWC + 0.0016TK − 0.050TP	0.178 ***
40–60	Pathotrophic	β-diversity	0.899 + 0.0007AK + 0.0007AN − 0.006AP − 0.033TN	0.167 ***
		Turnover	0.714 + 0.0002AK − 0.0021TGB	0.072 *
		Nestedness	0.157 + 0.0008AN + 0.0015TGB + 0.0055TN + 0.0015AP	0.060 *

## Data Availability

The data presented in this study are available on request from the corresponding author due to commercial and intellectual property restrictions. This dataset contains proprietary information related to “A Soil Microbial Method for Characterizing Forest Succession Health,” which involves a patent currently under application.
